# Physical activity estimated by osteogenic potential and energy expenditure has differing associations with bone mass in young adults: the raine study

**DOI:** 10.1007/s11657-022-01100-1

**Published:** 2022-04-18

**Authors:** Carrie-Anne Ng, David Scott, Marc Sim, Kun Zhu, Aris Siafarikas, Nicolas H. Hart, Jocelyn Tan, Paola Chivers

**Affiliations:** 1 Department of Medicine, School of Clinical Sciences at Monash Health, Monash University, Clayton, VIC 3168 Australia; 2 Institute for Physical Activity and Nutrition, School of Exercise and Nutrition Sciences, Deakin University, Burwood, VIC Australia; 3 Institute for Nutrition Research, School of Medical and Health Sciences, Edith Cowan University, Joondalup, WA Australia; 4 Medical School, The University of Western Australia, Perth, WA Australia; 5 Western Australian Bone Research Collaboration, Perth, WA Australia; 6 Department of Endocrinology and Diabetes, Sir Charles Gairdner Hospital, Perth, WA Australia; 7 School of Medical and Health Sciences, Edith Cowan University, Joondalup, WA Australia; 8 Department of Endocrinology and Diabetes, Perth Children’s Hospital, Perth, WA Australia; 9 Telethon Kids Institute for Health Research, Perth, WA Australia; 10 Institute for Health Research, The University of Notre Dame Australia, Fremantle, WA Australia; 11 Caring Futures Institute, College of Nursing and Health Sciences, Flinders University, Adelaide, South Australia Australia; 12 School of Nursing, Faculty of Health, Queensland University of Technology, Brisbane, QLD Australia; 13 School of Nursing, Midwifery, Health Sciences and Physiotherapy, The University of Notre Dame Australia, Fremantle, WA Australia

**Keywords:** Physical activity, Bone mineral density, DXA, Peak bone mass, Population study

## Abstract

***Summary*:**

Ground impacts during physical activity may be important for peak bone mass. We found differences in how energy expenditure and impact scores estimated from a physical activity questionnaire related to bone health in young adults. Using both estimate types can improve our understanding of the skeletal benefits of physical activity.

**Purpose:**

It is unclear whether mechanical loading during physical activity, estimated from physical activity questionnaires which assess metabolic equivalents of task (METs), is associated with skeletal health. This longitudinal study investigated how physical activity loading scores, assessed at ages 17 and 20 years, (a) compares with physical activity measured in METs, and (b) is associated with bone mass at age 20 years.

**Methods:**

A total of 826 participants from the Raine Study Gen2 were assessed for physical activity energy expenditure via the International Physical Activity Questionnaire (IPAQ) at age 17 and 20 years. Loading scores (the product of peak force and application rate) per week were subsequently estimated from the IPAQ. Whole-body and appendicular bone mineral density (BMD) at age 20 years were assessed by dual-energy X-ray absorptiometry.

**Results:**

Bland–Altman minimal detectable difference for physical activity Z-

scores at age 17 and 20 years were 1.59 standard deviations (SDs) and 1.33 SDs, respectively, greater than the a priori minimal clinically important change of 0.5 SDs. Loading score, but not IPAQ score, had significant positive associations with whole-body and leg BMD after adjustment for covariates (β = 0.008 and 0.012 g/cm^2^, respectively, for age 17 and 20 years loading scores). IPAQ score at age 20 years, but not loading score, had a significant positive association with arm BMD (β = 0.007 g/cm^2^).

**Conclusion:**

This study revealed disagreement in associations of self-reported METs and loading score estimates with bone health in young adults. Coupling traditional energy expenditure questionnaire outcomes with bone-loading estimates may improve understanding of the location-specific skeletal benefits of physical activity in young adults.

**Supplementary Information:**

The online version contains supplementary material available at 10.1007/s11657-022-01100-1.

## Introduction

Maximising peak bone mass attainment by young adulthood is important for long-term skeletal health. Specifically, peak bone mass is estimated to be six times more influential on the development of osteoporosis than other well-established risk factors, including age of menopause or rate of bone loss [1]. Optimising bone accrual during the critical peri-adolescent growth period may thus be of greatest significance in preventing fractures as we age [2, 3]. The positive influence of physical activity on peak bone mass is well recognised, but recommendations on the optimal type, dose, and frequency of activity remain unclear [3, 4].

Physical activities with a combination of high and rapid impact, multi-directional loading, and weight-bearing have the most significant physiological effects on bone structure [5]. Targeted high-impact exercise in randomised controlled trials results in increased bone mineral density (BMD) and bone strength during the prepubertal and peripubertal stages [6, 7]. However, in determining the skeletal benefits of habitual physical activity, observational studies have commonly utilised traditional physical activity questionnaires with calculations based on metabolic equivalents of task (METs), such as the International Physical Activity Questionnaire (IPAQ) [4, 8]. Such methodologies fail to capture key characteristics of osteogenesis during specific physical activities, specifically mechanical load magnitude and application rate.

To better understand associations between physical activity and bone health, activities should be quantified by the intensity and application rate of ground reaction forces generated, based on underlying principles of the *osteogenic index* [9, 10]. Taking such principles into account, the Bone-specific Physical Activity Questionnaire (BPAQ) was developed. The BPAQ utilises measured effective load ratings for a range of physical activities based on the intensity and application rate of ground reaction forces exerted on the lower limb [10]. Cross-sectional studies using the BPAQ have since demonstrated that osteogenic physical activity has location-specific benefits for tibial shaft microarchitecture in children and young adults [11, 12].

However, there is limited evidence favouring such bone loading questionnaires over METs estimates when assessing bone outcomes. In older men, bone loading scores (derived from METs-based questionnaires), but not METs estimates themselves, were associated with greater maintenance of BMD over several years [13] and also with higher bone quality compared to total time spent in physical activity [14]. Similar adaptations of METs-based questionnaires have been undertaken in younger adults which likewise revealed positive associations between higher loading and bone mass and microarchitecture [15–18]. However, in these studies, few direct comparisons with energy estimates from the original questionnaire were made and as such it is unclear whether calculating bone loading scores provides additional insights into the effects of physical activity on bone health.

Effects of higher-impact physical activity in young adults who are in the maintenance phase of peak bone mass, estimated to occur after age 20 years [19], are also unclear. Of the few interventional studies conducted in this age group, improvements in bone mass from high-impact exercise were less marked compared to younger participants [3]. Furthermore, detraining in young adulthood may lead to a loss of skeletal benefits from physical activity due to bone remodelling [20]. Thus, the aims of this study were to: (a) compare energy expenditure and loading intensity estimated from a self-administered physical activity questionnaire and (b) determine whether participation in physical activity with higher loading intensities and rates assessed at ages 17 and 20 years are associated with bone mass at age 20 years.

## Materials and methods

### Study design

This study included data of the offspring (Gen2) of the Raine Study Gen1 participants. Pregnant women (n = 2900) were initially recruited from antenatal clinics at King Edward Memorial Hospital for Women in Perth, Western Australia, between 1989 and 1991. The resulting 2868 live born children underwent follow-up assessments at ages 1, 2, 3, 5, 8, 10, 14, 17, 20, and 22 years and were broadly representative of the Western Australian population [21]. The Raine Study Gen2 design has been described in detail elsewhere [21]. Written informed consent was obtained at each follow-up from parents or participants as appropriate for age. The original study and follow-ups were approved by the institutional ethics committees of King Edward Memorial Hospital, Princess Margaret Hospital for Children, the University of Western Australia, and Curtin University. This study was approved by The University of Notre Dame Australia (2020-094F), Edith Cowan University (2020–01705-SIM), and Monash University (25205) institutional human research ethics committees.

**Fig. 1:**
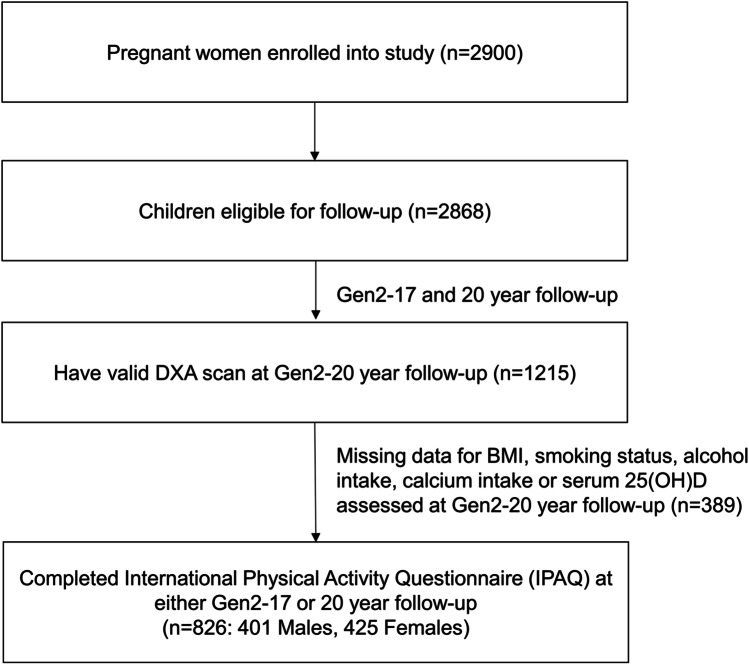
Raine study Gen2 participation flow diagram

The Raine Study Gen2–20-year follow-up methodology has been previously described according to investigations with the IPAQ [22], dual-energy X-ray absorptiometry (DXA), and vitamin D status [23, 24]. 1348 participants attended the physical assessment component at the Gen2–20-year follow-up. Of these, 73 participants did not undergo a DXA scan and a further 92 did not have a valid DXA scan due to the presence of artefacts in the region of interest or because participants could not fit in the scanning area. Of the participants who had a valid DXA scan, further complete data for body mass index (BMI), smoking habits, alcohol consumption, dietary calcium intake, and serum 25-hydroxyvitamin D (25(OH)D) were available for 826 participants (Fig. 1). These participants also completed the IPAQ at either the Gen2–17- or Gen2–20-year follow-ups, with 629 completing the questionnaire at both time points. Compared to the Raine study participants who attended the Gen2–20-year follow-up but were excluded (39%), the participants in the present study did not differ significantly by physical activity or bone parameters, except for arm bone mineral content (BMC) and BMD which were significantly lower among those included (Supplementary Table 1).

### Physical activity

Physical activity in the past 7 days was assessed via the IPAQ, previously validated with objective measures of physical activity [25]. The long IPAQ form was self-administered at the Raine Study Gen2–17-year follow-up while the short form was self-administered at the Gen2–20-year follow-up. This was likely due to a qualitative preference of the short form, and no observed differences in the reliability and validity of both forms [26]. In the short version, participants reported the frequency, in days, and duration, in hours and minutes per day, of walking, moderate activity, and vigorous activity. The long form further assessed the frequency and duration of these activity intensities within five subdomains: occupational activity, leisure activity, active transport, housework, and yard work (Supplementary Table 2). Data cleaning, processing and the categorising of low, moderate and high activity participation were performed according to guidelines by the IPAQ research committee [26]. The resulting IPAQ scores (MET-min/wk) were calculated as frequency × duration × MET estimate, summed across physical activity domains for the short form, or subdomains for the long form (Supplementary Table 2).

To assess the osteogenic potential of physical activity, effective load ratings (ELRs) were used instead of MET estimates (Supplementary Table 2), similar to previous work [13]. ELRs were previously determined following the principles of estimating intensity and application rate of ground reaction forces used in the BPAQ [10]. Briefly, the BPAQ estimates peak vertical ground reaction force and the rate of force application of the fundamental actions of an activity using a force platform. The ELR of a physical activity is the product of the peak force and application rate of the fundamental actions composing the activity, and aggregate values for impact intensity categories were used [27]. Loading scores (ELR/wk) were calculated as frequency × ELR, also summed across physical activity domains and subdomains. Days of physical activity per week, rather than minutes, were used in this equation as osteogenesis is reported to be enhanced by number of sessions rather than the duration of individual sessions [9].

To allow for comparability between short and long forms between the two time points, an adapted calculation was applied to the long form where physical activity subdomains were assigned the same MET estimate or ELR as its associated domain (Supplementary Table 2).

### Whole-body DXA

Whole-body DXA scans were performed at the Raine Study Gen2–20-year follow-up visit using the Norland XR-36 densitometer (Norland Medical Systems, Inc., Fort Atkinson, WI, USA) according to manufacturer-recommended procedures [23, 24]. Scan analysis using the built-in machine software (version 4.3.0) provided estimates of BMC (g) and areal BMD (g/cm^2^) for the whole body (including head), legs and arms. Whole-body fat percentage (%) and lean mass (kg) were also assessed from whole-body scans. All analyses were checked for consistency by the same researcher. Daily calibration was performed prior to each scanning session, and the interscan coefficient of variation (CV) was less than 2.0% at standard speed.

### Anthropometric, sociodemographic, and lifestyle measures

At the Raine Study Gen2–20-year follow-up visit, height was measured to the nearest 0.1 cm with a stadiometer (Seca 202, Hanover, MD) and weight was measured to the nearest 0.1 kg using an automatic electronic scale (Personal Precision scales UC‐321; A&D Company). Participants wore light clothing without shoes during measurements. BMI was calculated as body mass (kg)/ squared height (m^2^). Usual dietary intake was assessed by the Dietary Questionnaire for Epidemiological Studies (DQES V2), a validated 74-item semi-quantitative food frequency questionnaire developed by the Cancer Council of Victoria [28]. The data collected by DQES v2 were used to calculate dietary calcium intake (mg/day) and presence of alcohol beverage intake (never or “sometimes”). Smoking was assessed by a questionnaire via the question “Do you currently smoke cigarettes/cigars?” and participants were categorised as smokers or non-smokers.

### 25(OH)D

Fasting venous blood samples were collected at the Raine Study Gen2–20-year follow-up and stored at -80 °C until analysed. Serum 25(OH)D_2_ and 25(OH)D_3_ concentrations were measured by RMIT Drug Discovery Technologies using isotope dilution liquid chromatography–tandem mass spectrometry (LC–MS/MS). As blood samples were collected year round, the seasonal component was removed from serum 25(OH)D concentrations according to published formulae [29]. Total serum 25(OH)D was the summation of deseasonalised 25(OH)D_2_ and 25(OH)D_3_. The CVs for 25(OH)D_3_ were 5.8% at 28 nmol/l, 5.2% at 80 nmol/l and 9.2% at 188 nmol/l, and the CVs for 25(OH)D_2_ were 7.9% at 25 nmol/l, 6.6% at 75 nmol/l and 10.4% at 185 nmol/l.

### Statistical analyses

Characteristics of participants were summarised with descriptive statistics and compared across groups based on tertile cut points of loading score at the Raine Study Gen2–20-year follow-up, using one-way ANOVA or Kruskal–Wallis tests for continuous variables and Chi-square tests for categorical variables. Normality of continuous variables was assessed via histograms. Bonferroni post hoc tests or Dunn’s post-test were performed for these analyses. Wilcoxon signed-rank tests compared IPAQ and loading scores from the Gen2–17- to Gen2–20-year follow-ups.

Spearman’s correlation assessed the relationship between IPAQ scores and ELRs at each follow-up time point. While correlation can describe the strength of linear relationships, it does not necessarily suggest comparability or agreement [30]. Hence, to estimate agreement between the physical activity measures, Bland–Altman plots were constructed separately at Gen2–17- and Gen2–20-year follow-ups, where differences between Z-score transformed IPAQ and loading scores were plotted against their averages. The 95% confidence interval (CI) limits of agreement were calculated as mean bias ± 1.96 standard deviation (SD) of the differences and represent 95% of the difference between the two scores. The minimal detectable change (MDC) was then obtained, defined as one-half the limit of agreement width, and is the smallest change between IPAQ and loading scores independent of measurement error. A MDC greater than an a priori minimal clinically important change (MCIC) of 0.5 SDs [31] would indicate clinically important disagreement between IPAQ and loading scores. To detect proportional bias, which may occur when the differences in Z-IPAQ and loading scores change in proportion to their average, linear regression was additionally performed.

To examine potential non-linearity, a likelihood ratio test was first used to compare nested models with and without the nonlinear terms for IPAQ and loading scores. For linear associations, generalised linear models compared bone and body composition parameters with standardised IPAQ and loading scores at the Raine Study Gen2–17- and Gen2–20-year follow-ups, and standardised change in IPAQ and loading scores between the two time points. Models were presented as: Model 1 which adjusted for sex and BMI at Gen2–20-year follow-up and Model 2 which included Model 1 + smoking status, alcohol consumption, dietary calcium intake and serum 25(OH)D at Gen2–20-year follow-up. Self-rated health and well-being assessed via the 12-item health survey was not a significant predictor of outcome variables in any model and was not included as a covariate. A predictive equation was generated to estimate whole-body BMD for the maximum loading score able to be detected by the questionnaire at age 20 years. To compare the goodness of fit of Models 1 and 2, the Akaike Information Criterion (AIC) was used, where the model with the smaller AIC values was considered a better fit [32].

For analyses between physical activity assessed at the Gen2–17-year follow-up and bone parameters, the original long-form physical activity scores were used for comprehensiveness. For analyses of change in physical activity, the adapted long-to-short form of scores assessed at Gen2–17-year follow-up was used to allow for comparability between the time points. Further analysis examining interaction terms for sex was conducted to determine if associations differed between males and females. For significant observed interactions, subsequent analyses were performed separately for each gender to investigate where differences lay. To examine if observed associations were independent of intensity levels or loading scores of physical activity, analyses were performed where IPAQ score was added as a covariate in loading score analyses, and vice versa. For this analysis, collinearity between IPAQ and loading scores was assessed in using the variance inflation factor (VIF), with a value of > 4 to be evidence of collinearity [33].

Statistical analyses were performed with SPSS IBM software (version 25; IBM, Chicago, IL, USA), and graphs were generated in R (version 4.0.3; R Foundation for Statistical Computing). Statistical significance was defined as p < 0.05 (2-tailed).

## Results

Descriptive variables of included participants at the Raine Study Gen2–20-year follow-up visit are presented in Table 1. Participants within the highest tertile of loading score were more likely to be male and had higher daily dietary calcium intake and serum 25(OH)D levels compared to those in the lowest tertile. Lean mass, arm BMC, whole-body, and leg BMC and BMD were also significantly higher among participants in the highest tertile compared to the middle and lowest tertile, with the converse observed for total fat percentage. There were no significant differences in age, BMI, smoking status, or alcohol consumption between tertiles.

**Table 1:** Characteristics of the raine study participants at Gen2–20-year follow-up according to tertiles of loading score

			Tertiles of loading score at Gen2–20-year follow-up (N = 823)					
	Included into analysis		Lowest < 70.1 ELR/wk		Middle 70.1–220.7 ELR/wk		Highest > 220.7 ELR/wk	
N	826		274		274		275	
	Mean or %	SD	Mean or %	SD	Mean or %	SD	Mean or %	SD
Age	19.96	0.44	19.92	0.43	19.97	0.47	19.98	0.41
Sex (% of males)	48.5		32.5 ^b,c^		46.4 ^a,c^		66.9 ^a,b^	
BMI (kg/m^2^)	23.88	4.30	23.45	4.30	23.80	4.14	24.31	4.33
Smoker (%)	13.8		14.2		12.8		14.2	
Alcohol consumer (%)	92.9		92.0		92.7		94.2	
Calcium Intake (mg/day)	903.9	409.6	816.9 ^c^	376.5	886.7 ^c^	395.5	1003.0 ^a,b^	427.8
Serum 25(OH)D (nmol/L)	73.68	23.51	69.10 ^c^	23.09	71.99 ^c^	23.25	79.91 ^a,b^	22.74
Whole-body total fat (%)	30.85	12.52	35.20 ^b,c^	12.06	31.36 ^a,c^	11.54	25.87 ^a,b^	12.15
Whole-body lean mass (kg)	46.28	12.16	40.94 ^b,c^	9.93	45.41 ^a,c^	11.37	52.43 ^a,b^	12.12
Whole-body BMC (g)	2938.0	459.2	2804.8 ^b,c^	395.4	2896.2 ^a,c^	458.3	3110.9 ^a,b^	467.4
Whole-body BMD (g/cm^2^)	1.072	0.109	1.042 ^b,c^	0.994	1.064 ^a,c^	0.110	1.110 ^a,b^	0.107
Whole-body Z-score	0.14	1.34	-0.29 ^b,c^	1.17	-0.08 ^a,c^	1.35	0.54 ^a,b^	1.35
Arms BMC (g)	374.1	83.5	347.3 ^b,c^	71.1	365.7 ^a,c^	82.3	409.3 ^a,b^	84.3
Arms BMD (g/cm^2^)	0.784	0.091	0.765 ^c^	0.086	0.775 ^c^	0.090	0.811 ^a,b^	0.091
Legs BMC (g)	1045.4	197.4	979.1 ^b,c^	172.0	1032.0 ^a,c^	197.0	1124.7 ^a,b^	194.5
Legs BMD (g/cm^2^)	1.166	0.134	1.124 ^b,c^	0.122	1.157 ^a,c^	0.135	1.217^a,b^	0.129
	Median	IQR	Median	IQR	Median	IQR	Median	IQR
IPAQ Score (MET-min/week)	2466.0	838.0 —4920.0	693.0 ^b,c^	99.0 —1971.5	2578.0 ^a,c^	1440.0 — 4212.0	6000.0 ^a,b^	2853.0 — 9390.0
Loading Score (ELR/week)	152.5	43.7 — 274.5	14.8 ^b,c^	2.0 — 43.7	152.5 ^a,c^	111.2 — 181.4	318.1 ^a,b^	263.7 —373.1

^a^ Significant difference to tertile 1

^b^ Significant difference to tertile 2

^c^ Significant difference to tertile 3 (Bonferroni post hoc tests or Dunn’s post-test)

Abbreviations: ELR, effective load rating; SD, standard deviation; BMI, body mass index; 25(OH)D, 25-hydroxyvitamin D; BMC, bone mineral content; BMD, bone mineral density; IQR, inter-quartile range; IPAQ, International Physical Activity Questionnaire; MET, metabolic equivalent of task

**Fig. 2:**
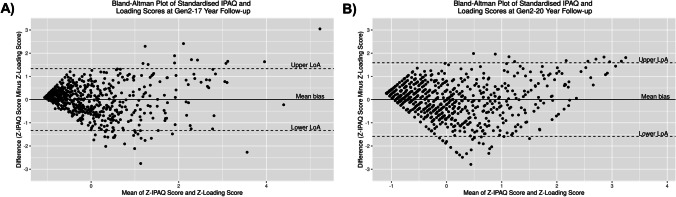
Bland–Altman plots for standardised IPAQ and loading scores at: **A)** Gen2–17-year and **B)** Gen2–20-year follow-ups. The x-axis displays the mean of Z-score transformed IPAQ and loading scores and y-axis displays the difference of the two estimates. The central line represents the mean bias (intermethod difference), which is 0 as Z-scores were used. The dashed lines indicate the 95% limits of agreement. Abbreviations: IPAQ, International Physical Activity Questionnaire; LoA, limits of agreement

From the Gen2–17-year to Gen2–20-year follow-up, median self-reported physical activity scores decreased from 3070 (IQR: 1140.5 – 5602.5) to 2400 (831.3 – 4773.0) MET-min/wk for IPAQ scores (p < 0.001), with a reduction in moderate and high activity participation from 31.8% and 60.9% to 24.5% and 48.5%, respectively. Loading scores also decreased from 154.1 (IQR: 54.7 – 289.4) to 152.9 (54.9 – 263.7) ELR/wk (p < 0.001). IPAQ scores were positively correlated with loading score at both Gen2–17-year (r_s_ = 0.75, p < 0.001) and Gen2–20-year follow-ups (r_s_ = 0.64, p < 0.001). Figure 2 presents Bland–Altman plots at both follow-ups. The lower and upper limits of agreement were -1.33 and 1.33, respectively, at Gen2–17-year follow-up, and -1.59 and 1.59, respectively, at Gen2–20-year follow-up. As the average of the standardised scores increased, the dispersion of the differences increased. At each follow-up, the MDC was greater than the a priori MCIC of 0.5, indicating clinically relevant disagreement between the two scores. Linear regression did not reveal proportional bias for both comparisons (both p = 1.000).

The multivariable-adjusted relationship between whole-body BMC and BMD, and IPAQ and loading scores at Gen2–17- or Gen2–20-year follow-up, or their change, were of a linear nature (p for non-linearity > 0.054 in Model 2**;** Supplementary Fig. 1). Thus, generalised linear models determined associations of IPAQ and loading scores at Gen2–17-year (Table 2) and Gen2–20-year follow-ups (Table 3), and their changes between follow-ups (Supplementary Table 3) with DXA-derived bone and body composition parameters at Gen2–20-year follow-up. Loading score at Gen2–17-year follow-up was positively associated with all bone parameters and lean mass, and negatively associated with total fat percentage in Model 1 (all p ≤ 0.010) and 2 (all p < 0.039), except for whole-body BMC which was not significant after adjustment for multiple confounders in Model 2. IPAQ score was positively associated with arms BMC (p = 0.039) and BMD (p < 0.001) and negatively associated with total fat percentage (p = 0.002) in Model 2.

**Table 2:** Associations between DXA-derived measures at Gen2–20-year follow-up per standard deviation increase in IPAQ and loading scores at Gen2–17-year follow-up

	Model 1		Model 2	
	IPAQ score	Loading score	IPAQ score	Loading score
Whole-body				
BMC (g)	28.94 (-0.89, 58.77)	41.25* (9.72, 72.77)	15.88 (-11.92, 43.67)	24.03 (-5.74, 53.80)
BMD (g/cm^2^)	0.010* (0.002, 0.017)	0.011** (0.004, 0.019)	0.007 (0.000, 0.013)	0.008* (0.000, 0.015)
Arms				
BMC (g)	7.25** (2.17, 12.33)	8.58** (3.47, 13.70)	4.93* (0.25, 9.60)	5.89* (1.05, 10.73)
BMD (g/cm^2^)	0.013** (0.007, 0.019)	0.011** (0.005, 0.017)	0.011** (0.005, 0.017)	0.008** (0.002, 0.014)
Legs				
BMC (g)	9.55 (-2.58, 21.67)	19.47** (6.47, 32.47)	4.95 (-6.78, 16.69)	13.60* (1.07, 26.13)
BMD (g/cm^2^)	0.010* (0.001, 0.019)	0.017** (0.007, 0.026)	0.007 (-0.002, 0.015)	0.012** (0.004, 0.021)
Total Fat (%)	-1.03** (-1.55, -0.52)	-1.31** (-1.81, -0.80)	-0.73** (-1.20, -0.26)	-0.97** (-1.44, -0.50)
Total Lean Mass (kg)	0.81** (0.21, 1.40)	1.18** (0.62, 1.75)	0.51 (-0.04, 1.05)	0.79** (0.26, 1.32)

Data presented as β coefficients (95% confidence interval). Bolded values are significant at p < 0.05* or p < 0.01**

Model 1 adjusted for sex and BMI at Gen2–20-year follow-up

Model 2 adjusted for sex, BMI, smoking, alcohol, calcium intake and serum 25(OH)D at Gen2–20-year follow-up

Abbreviations: DXA, dual-energy x-ray absorptiometry; IPAQ, International Physical Activity Questionnaire; BMC, bone mineral content; BMD, bone mineral density; BMI, body mass index; 25(OH)D, 25-hydroxyvitamin D

**Table 3:** Associations between DXA-derived measures per standard deviation increase in IPAQ and loading scores at Gen2–20-year follow-up

	Model 1		Model 2	
	IPAQ score	Loading score	IPAQ score	Loading score
Whole-body				
BMC (g)	53.78** (28.71, 78.86)	58.65** (33.72, 83.58)	34.48** (10.14, 58.82)	34.51** (9.44, 59.58)
BMD (g/cm^2^)	0.009** (0.003, 0.015)	0.013** (0.007, 0.019)	0.005 (-0.001, 0.010)	0.008* (0.001, 0.014)
Arms				
BMC (g)	11.75** (7.43, 16.08)	10.24** (5.96, 14.53)	8.64** (4.48, 12.79)	6.36* (2.05, 10.67)
BMD (g/cm^2^)	0.010** (0.004, 0.015)	0.009** (0.004, 0.014)	0.007* (0.001, 0.012)	0.005 (0.000, 0.011)
Legs				
BMC (g)	22.04** (11.62, 32.45)	27.07** (16.74, 37.39)	15.10** (4.85, 25.36)	18.74** (8.25, 29.24)
BMD (g/cm^2^)	0.011** (0.004, 0.019)	0.018** (0.010, 0.025)	0.006 (-0.001, 0.014)	0.012** (0.004, 0.020)
Total Fat (%)	-1.34** (-1.76, -0.92)	-1.94** (-2.36, -1.53)	-0.91** (-1.32 -0.49)	-1.49** (-1.90, -1.07)
Total Lean Mass (kg)	1.55** (1.10, 2.01)	1.92** (1.49, 2.34)	1.09** (0.66, 1.53)	1.38** (0.97, 1.80)

Data presented as β coefficients (95% confidence interval). Bolded values are significant at p < 0.05* or p < 0.01**

Model 1 adjusted for sex and BMI at Gen2–20-year follow-up

Model 2 adjusted for sex, BMI, smoking, alcohol, calcium intake and serum 25(OH)D at Gen2–20-year follow-up

Abbreviations: DXA, dual-energy x-ray absorptiometry; IPAQ, International Physical Activity Questionnaire; BMC, bone mineral content; BMD, bone mineral density; BMI, body mass index; 25(OH)D, 25-hydroxyvitamin D

At Gen2–20-year follow-up, IPAQ and loading scores were positively associated with all bone parameters and total lean mass, and negatively associated with total fat percentage in Model 1 (all p < 0.010) (Table 3). After further adjustment in Model 2, the association between IPAQ score and whole-body BMD and leg BMD, and between loading score and arm BMD was attenuated. IPAQ score had greater standardised effects with arm BMC and BMD than loading score, while loading score had greater standardised effects with whole-body and leg BMC and BMD, total fat percentage, and lean mass. Loading score was positively associated with whole-body BMD (p = 0.017), and the predictive equation was:

$$\begin{aligned}Whole-body\;BMD\left(g/{cm}^2\right)&=0.698+0.096\;\left(\times1\;if\;male\right)+0.011\;\left(BMI\right)+0.004\;(\times\;1\;if\;non-smoker)\\& -0.035\;\left(\times\;1\;if\;no\;alcohol\;consumption\right)+0.001\;\left(Serum\;25(OH)D)\right)\\&+0.000\;\left(Calcium\;intake\right)+0.008\;\left(Z-score\;of\;loading\;score\right)\end{aligned}$$

As an example, for a male non-smoker who does not consume alcohol with mean values for serum 25(OH)D and calcium intake, and who had a maximal loading score (483.14 ELR/wk; Z-score = 2.34) at the Gen2–20-year follow-up, the equation would be:

$$=0.698+0.096+0.011\left(23.88\right)+0.004-0.035+0.001\left(73.68\right)+0.000\left(903.9\right)+0.008\left(2.34\right)$$

resulting in predicted whole-body BMD of 1.106g/cm^2^.

AIC values in Model 2 were lower than observed in Model 1 at both follow-ups for each outcome, indicating a better model fit. There were significant sex and IPAQ score interactions for arm BMD in Models 1 and 2 at Gen2–17-year follow-up, and Model 1 at Gen2–20-year follow-up. Separate analyses revealed significant positive associations between IPAQ score and arm BMD for males (β > 0.012 g/cm^2^) and non-significant associations for females (β < 0.001 g/cm^2^).

When loading score was added as a covariate to Model 2 in IPAQ score analyses, only arm BMD remained significantly positively associated with IPAQ score at Gen2–17-year follow-up (β = 0.010 g/cm^2^, 95% CI = 0.002, 0.018), and arm BMC remained significantly positively associated with IPAQ score at Gen2–20-year follow-up (β = 7.71 g, 95% CI = 2.35, 13.08). Conversely, when IPAQ score was added as a covariate, only leg BMC and BMD remained significantly positively associated with loading score at both Gen2–17-year (β = 20.86 g, 95% CI = 4.37, 37.35 for leg BMC and β = 0.016 g/cm^2^, 95% CI = 0.004, 0.027 for leg BMD) and Gen2–20-year follow-ups (β = 14.89 g, 95% CI = 1.20, 28.57 for leg BMC and β = 0.013 g/cm^2^, 95% CI = 0.004, 0.023 for leg BMD). In this adjustment, VIF values for IPAQ and loading score, respectively, were 2.34 and 2.31 at Gen2–17-year follow-up, and 1.87 and 1.95 at Gen2–20-year follow-up.

Changes in IPAQ and loading score from Gen2–17- to Gen2–20-year follow-ups were not significantly associated with any bone or body composition measures (Supplementary Table 3).

## Discussion

This study used a novel approach to estimate bone loading from an energy expenditure-based physical activity questionnaire and investigated its association with bone parameters in young adults. We found clinically important disagreement between loading scores and energy expenditure measured by IPAQ at both Gen2–17- and Gen2–20-year follow-ups. Participation in physical activity with higher loading scores was more strongly associated with greater whole-body and leg bone mass, while energy expenditure was positively associated with arm bone mass. However, there were no observed significant associations between change in loading or IPAQ scores and bone parameters.

Bland–Altman analyses revealed wide limits of agreement greater than the MCIC of 0.5 SD, which is a clinically important threshold in discriminating between self-reported health-related measures [31]. This indicates that loading scores and IPAQ scores cannot be used interchangeably [34] and confirms our hypothesis that METs are insufficient in identifying bone-relevant mechanical loading. However, as neither score reflects objective means of measuring physical activity, we can only make relative comparisons independent of the subjective nature of the questionnaire. Regardless, such differences have previously been demonstrated in young adults whereby a weak, non-significant correlation between METs/week and BPAQ score of the past one year was reported (r = -0.26) [10]. In the current study, the moderate to strong positive correlation between IPAQ and loading scores may have been attributed to reduced variation at smaller magnitudes, observed by a narrower dispersion at lower scores in the plots. Indeed, it may be difficult to differentiate the mechanical loading and cardiometabolic components of physical activity in relatively sedentary individuals. Further generalisability of our findings is limited by a lack of correlation or agreement analyses in studies of bone loading scores [16, 17, 35]. Relevant past findings may also have been confounded by different observed self-report timeframes, such as in a study of young adult females, where energy expenditure over the past week was not correlated with lifetime bone loading scores (r = 0.02) [12].

We observed that loading scores, but not IPAQ scores, at both Gen2–17- and Gen2–20-year follow-ups, were associated with whole-body and leg BMD in the fully adjusted model. The lack of significant associations between physical activity scores and whole-body and leg BMC at Gen2–17-follow-up, compared to that at Gen2–20, may be because taller participants in the Raine Study may not have yet attained peak bone mass [24]. Indeed, adjusting for height rather than BMI in the models resulted in positive significant associations between IPAQ and loading scores and BMC at all sites (data not shown). β values between standardised loading scores and BMC and BMD at these sites were also higher than that of IPAQ scores. These results correspond with findings from a systematic review in young adults, whereby studies assessing weight-bearing physical activities demonstrated more consistent positive associations with bone mass compared to when physical activity was quantified by energy estimates [4]. However, few direct comparisons of these distinct physical activity types in the same population have been made. Notably, in the Amsterdam Growth and Health Longitudinal Study (AGAHLS), the mechanical component of physical activity in young adulthood (ages 21–27 years), but not the metabolic component, was associated with lumbar spine and femoral neck BMD [18, 35]. Interestingly, both components of physical activity during adolescence (ages 13–16 years) in this study were not associated with BMD at either site [18, 36], suggesting that loading during the years of peak bone mass may be more conducive for osteogenesis, or that bone structural changes may have occurred that were undetected by DXA scans.

However, the pre- and peri-pubertal periods, where there is high linear growth of bone, have been well established to be optimally responsive to mechanical loading [7]. Our findings may thus be attributed to maintenance of participation in physical activity of high to moderate impact from adolescence to young adulthood. Indeed, almost half of the Raine Study Gen2 participants were reported to have consistent organised sport participation trajectories, and this group had greater peak BMC than sport dropouts [37]. However, the nature of these sports is unclear as physical activity prior to age 17 years was assessed in the Raine Study by a single polar (yes/no) question about participation in organised sport outside of school hours. In the current study, no significant association was observed between change in either loading or IPAQ scores and bone mass. This may be because the effects of physical activity at age 17 years on bone may be indistinguishable to that at age 20 years. When IPAQ or loading score at 17 years was included in the models, an increase in IPAQ scores over three years was significantly positively associated with BMC at all sites, while an increase in loading scores was only similarly associated with leg BMC (data not shown). This suggests that while young adults can begin participation in more metabolically intense activities in young adulthood to improve overall bone mass, a more consistent participation in impact physical activity from earlier in life may be required. Longitudinal studies including the AGAHLS have commonly defined specific physical activity time periods such as adolescence or young adulthood when investigating their skeletal effects [16–18, 36]. When trends of physical activity were evaluated, sustained high-impact activity from adolescence to adulthood was associated with BMD at clinically relevant sites in males [17]. As such, the current study’s short observatory period of physical activity may have limited us in explaining our findings.

From our predictive equation example, the estimated whole-body BMD of 1.106 g/cm^2^ in an average male who achieves a maximal loading score is higher than the mean BMD by 0.31 SD. A previous study in Raine Study Gen2 participants reported a comparable increase of 0.35 SD in whole-body BMD at age 20 years among those with consistently higher vitamin D status trajectory from age 6 years [23], suggesting the importance of lifestyle and physiological factors in influencing peak bone mass. Clinically, a 1 SD increase in peak bone mass can reduce osteoporotic fracture risk in later life by 50% [2]. In the current study, a maximal loading score would be achieved if one performs a combination of walking, and moderate and vigorous physical activity daily for at least 10 min. Participants who achieved this may have had more varied physical activity types with greater diversification of loading favourable for osteogenesis [38].

Location-specific skeletal effects of loading scores were apparent, where positive associations between loading scores and leg BMC and BMD were independent of IPAQ score. Similar findings were reported in the Gothenburg Osteoporosis and Obesity Determinants study, where higher physical activity peak strain score in young adults was associated with significantly greater BMD at the femoral neck and lumbar spine (10.5–14.0% difference with sedentary group) compared to at the radius (3.0% difference) [39]. Calculation of these peak strain scores applied ground reaction force principles like in our current method and placed greater emphasis on activities that involved strain to the lower limb, such as jumping [36]. As such, upward dissipation of forces may result only in observations of associations at the lower limb and axial sites. Indeed, we found that IPAQ scores were positively associated with arm BMC and BMD, with associations tending to remain significant after adjustment with loading score. Our observed associations were driven by males, whose bones may have sustained mechanosensitivity to physical activity after puberty compared to females [8]. In contrast, past studies demonstrate a lack of association between physical activity metabolic intensity and radius bone mass and microarchitecture, instead citing lean mass, body weight, or physical function as stronger predictors [40, 41]. However, the association between IPAQ scores at Gen2–17-year follow-up and arm BMD remained significant following adjustment for lean mass (data not shown), suggesting that the osteogenic effect of physical activity was not a function of local effects. It is possible that males in this study engaged in greater weight-bearing activities at the upper arm such as weightlifting and rugby [42], and perceived the intensity of such activities as moderate, which can disproportionately increase IPAQ scores relative to loading scores.

Despite these positive findings, this study has several limitations. The observational nature of the study prevents us from inferring causality, and the study was not designed to longitudinally observe the skeletal effects of physical activity types. As such, the long and short IPAQ forms were administered at the Gen2–17- and Gen2–20-year follow-ups, respectively. It has been reported that the two forms have poor agreement [43]. Our long-to-short-form adaptation intended to overcome this incompatibility, but this conversion has not been validated, and may have contributed to the lack of observed association between change in physical activity and bone mass. Self-reported physical activity intensity levels are also subject to recall bias, physical function, and individual interpretation. The latter has been a criticism of the IPAQ due to its ambiguous instructions, particularly describing moderate and vigorous physical activity as making one “breathe harder than normal”, thus creating difficulties in differentiating between activities of varying intensities [44]. Such IPAQ questions were also designed to assess metabolic intensity, instead of mechanical loading. However, it is probably more unlikely that individuals are able to conceptualise and distinguish between moderate- and high-impact physical activity as these forms of activities are less familiar. Instead, past studies have extracted bone loading scores from physical activities recorded in free-text form [45]. This can be a time-consuming task in large cohorts, especially in a young population where types of physical activity can vary greatly. Our current approach may have simplified this process for ease of loading score calculation, but its accuracy and validity are unknown. We also did not examine bone mass at other clinically relevant skeletal sites, which may have achieved peak bone mass at varying stages and respond to mechanical loading differently [19], nor did we adjust for maturity due to insufficient data regarding the timing of puberty in our cohort.

Our novel approach may support retrospective re-analyses of existing datasets where peak bone mass is of interest. Coupling traditional energy expenditure questionnaire outcomes with bone-loading estimates may also improve understanding of the location-specific skeletal benefits of physical activity in young adults. In conclusion, our study revealed important disagreements in associations of loading intensity and energy expenditure from a self-administered physical activity questionnaire with peak bone mass in young adults, but limited relationships with change in physical activity measures from age 17 to 20 years.

## Supplementary Information

Below is the link to the electronic supplementary material.

Supplementary file1 (DOCX 3317 KB)
