# Supplementary material for: Physical activity estimated by osteogenic potential and energy expenditure has differing associations with bone mass in young adults: the raine study
Source: Arch Osteoporos. 2022 Apr 18;17(1):67. doi: 10.1007/s11657-022-01100-1 (PMC9013684; doi:10.1007/s11657-022-01100-1)
Supplement: Supplementary file 1 — Supplementary file1 (DOCX 3317 KB) [file 11657_2022_1100_MOESM1_ESM.docx]

*Supplementary Table 1 Characteristics of the Raine Study Participants at Gen2-20 Year Follow-Up Who Were Included and Excluded from the Current Analysis*

|  | **Included into Analysis** | | **Excluded from Analysis** | | **p-value** |
| --- | --- | --- | --- | --- | --- |
| N | 826 | | 522 | |  |
|  | Mean | SD | Mean | SD |  |
| **Age** | 19.96 | 0.44 | 20.09 | 0.48 | **<0.001*** |
| **Sex** (% of males) | 48.5 |  | 58.3 |  | **<0.001*** |
| **BMI** (kg/m^2^) | 23.88 | 4.30 | 24.88 | 5.62 | **0.001*** |
| **Smoker** (%) | 13.8 |  | 17.9 |  | 0.128 |
| **Alcohol consumer** (%) | 92.9 |  | 92.0 |  | 0.617 |
| **Dietary Calcium Intake** (mg/day) | 903.9 | 409.6 | 934.4 | 418.9 | 0.234 |
| **Serum 25(OH)D** (nmol/L) | 73.68 | 23.51 | 71.87 | 24.00 | 0.283 |
| **Whole-body total fat** (%) | 30.85 | 12.52 | 29.19 | 12.41 | **0.031*** |
| **Whole-body lean mass** (kg) | 46.28 | 12.16 | 49.04 | 11.97 | **<0.001*** |
| **Whole-body BMC** (g) | 2938.0 | 459.2 | 2981.3 | 462.5 | 0.126 |
| **Whole-body BMD** (g/cm^2^) | 1.072 | 0.109 | 1.081 | 0.115 | 0.190 |
| **Arm BMC** (g) | 374.1 | 83.5 | 386.4 | 86.2 | **0.017*** |
| **Arm BMD** (g/cm^2^) | 0.784 | 0.091 | 0.796 | 0.109 | **0.037*** |
| **Leg BMC** (g) | 1045.4 | 197.4 | 1060.3 | 201.4 | 0.225 |
| **Leg BMD** (g/cm^2^) | 1.166 | 0.134 | 1.172 | 0.142 | 0.464 |
|  | Median | IQR | Median | IQR |  |
| **IPAQ Score** (MET-min/week) | 2466.0 | 838.0 – 4920.0 | 2115.0 | 624.5 – 4923.0 | 0.230 |
| **Loading Score** (ELR/week) | 152.5 | 43.7 – 274.5 | 140.0 | 28.4 – 269.3 | 0.239 |
| Data presented as mean (SD), median (interquartile range) or %.  Abbreviations: ELR, effective load rating; SD, standard deviation; BMI, body mass index; 25(OH)D, 25-hydroxyvitamin D; BMC, bone mineral content; BMD, bone mineral density; IQR, inter-quartile range; IPAQ, International Physical Activity Questionnaire; MET, metabolic equivalent of task. | | | | | |

*Supplementary Table 2* *MET estimates and Effective Load Ratings Assigned to Physical Activity Domains and Subdomains in the Short and Long Forms of the IPAQ*

| **Physical Activity Domains and Subdomains ^a^** | **MET Estimate** | **Effective Load Rating** |
| --- | --- | --- |
| **Walking** | **3.30^b^** | **0.40** |
| Occupational | 3.30 | 0.40 |
| Leisure | 3.30 | 0.40 |
| Transport | 3.30 | 0.40 |
| **Moderate physical activity** | **4.00** | **13.62** |
| Occupational | 4.00 | 13.62 |
| Leisure | 4.00 | 13.62 |
| Moderate yard/garden work | 4.00 | 0.40 |
| Moderate household work | 3.00 | 0.40 |
| Vigorous yard/garden work | 5.50 | 3.00 |
| Transport – Cycling | 6.00 | 0.16 |
| **Vigorous physical activity** | **8.00** | **55.00** |
| Occupational | 8.00 | 55.00 |
| Leisure | 8.00 | 55.00 |
| ^a^ Activity domains were assessed in short form while activity subdomains were assessed in the long form.  ^b^ Bolded values were used in score calculation for the short form at age 20 years and adapted long-to-short form at age 17 years.  *Abbreviations: MET, metabolic equivalent of task; IPAQ, International Physical Activity questionnaire.* | | |

*Supplementary Table 3 Associations Between DXA-derived Measures at Gen2-20 Year Follow-up per Standard Deviation Change in IPAQ and Loading Scores (Gen2-20 Year Minus Gen2-17 Year Follow-up)*

|  | Model 1 | | Model 2 | |
| --- | --- | --- | --- | --- |
|  | **Change in IPAQ Score** | **Change in Loading Score** | **Change in IPAQ Score** | **Change in Loading Score** |
| Whole-body |  |  |  |  |
| BMC (g) | 16.46 (-12.44, 45.36) | 5.19 (-23.14, 33.52) | 12.44 (-14.92, 39.79) | 1.49 (-25.76, 28.74) |
| BMD (g/cm^2^) | -0.002 (-0.009, 0.005) | -0.001 (-0.008, 0.006) | -0.003 (-0.010, 0.004) | -0.002 (-0.009, 0.005) |
| Arms |  |  |  |  |
| BMC (g) | 2.58 (-2.01, 7.16) | -1.73 (-6.69, 3.24) | 2.11 (-2.24, 6.46) | -2.37 (-7.07, 2.33) |
| BMD (g/cm^2^) | -0.003 (-0.009, 0.002) | -0.004 (-0.009, 0.002) | -0.004 (-0.010, 0.002) | -0.004 (-0.010, 0.002) |
| Legs |  |  |  |  |
| BMC (g) | 7.61 (-3.93, 19.15) | 3.12 (-8.15, 14.38) | 6.16 (-5.11, 17.43) | 1.86 (-9.26, 12.97) |
| BMD (g/cm^2^) | -0.002 (-0.010, 0.006) | -0.002 (-0.010, 0.006) | -0.003 (-0.011, 0.005) | -0.003 (-0.011, 0.005) |
| Total Fat (%) | -0.02 (-0.50, 0.45) | 0.02 (-0.44, 0.48) | 0.05 (-0.40, 0.50) | 0.11 (-0.33, 0.54) |
| Total Lean Mass (kg) | 0.40 (-0.14, 0.95) | 0.20 (-0.32, 0.71) | 0.30 (-0.19, 0.80) | 0.11 (-0.37, 0.59) |
| Data presented as β coefficients (95% confidence interval). Bolded values are significant at p<0.05*.  Model 1 adjusted for sex and BMI at Gen2-20 year follow-up.  Model 2 adjusted for sex, BMI, smoking, alcohol, calcium intake and serum 25(OH)D at Gen2-20 year follow-up.  Abbreviations: DXA, dual-energy x-ray absorptiometry; IPAQ, International Physical Activity Questionnaire; BMC, bone mineral content; BMD, bone mineral density; BMI, body mass index; 25(OH)D, 25-hydroxyvitamin D. | | | | |

*
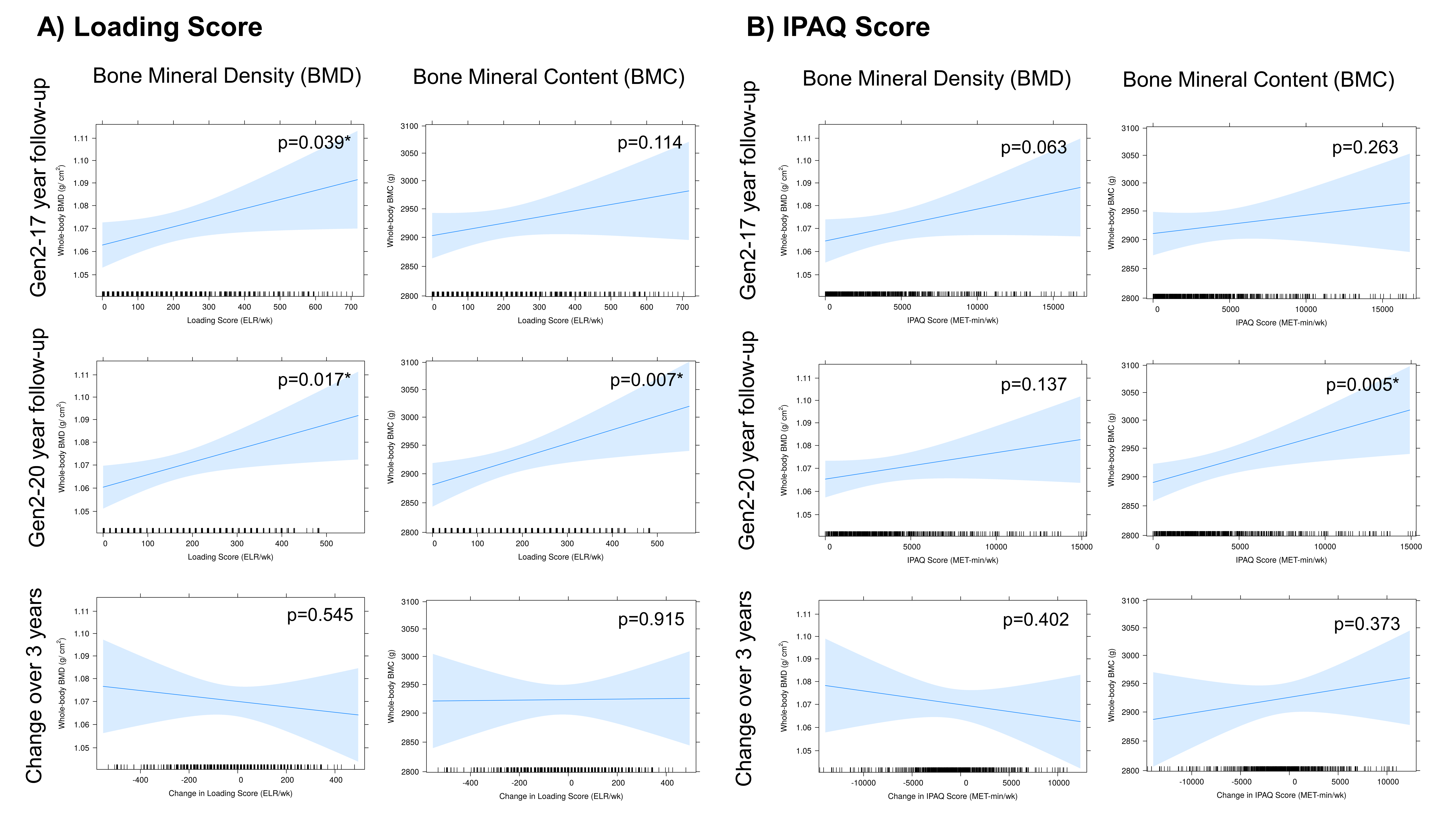
*

*Supplementary Figure 1. Graphic presentation of the multivariable-adjusted relationship between* ***A)*** *loading score and* ***B)*** *IPAQ score and whole-body BMC and BMD obtained by generalised regression models (Model 2, adjusted for sex, BMI, smoking, alcohol, calcium intake and serum 25(OH)D at Gen2-20 year follow-up). All relationships are of a linear nature (p for non-linearity>0.054). For visual simplicity, the x-axis was truncated at 3 standard deviations. Shaded area represents 95% confidence intervals. The rug plot along the bottom of each graph depicts each observation. p-values are of the generalised linear regression model. Abbreviations: IPAQ, International Physical Activity Questionnaire; BMC, bone mineral content; BMD, bone mineral density; ELR, effective load rating; BMI, body mass index; 25(OH)D, 25-hydroxyvitamin D.*
